# Encoding terahertz holographic bits with a computer-generated 3D-printed phase plate

**DOI:** 10.1038/s41598-024-56113-2

**Published:** 2024-03-06

**Authors:** E. Constable, J. Gospodaric, A. Pimenov

**Affiliations:** TU Wien, Solidstate spectroscopy, Vienna, Austria

**Keywords:** Terahertz optics, Optical data storage, Terahertz optics, Optical data storage

## Abstract

A sub-terahertz holographic image of a two-dimensional 576-bit data code is produced using a diffractive phase-plate element. The phase plate was designed using a modified Gerchberg-Saxton iterative algorithm to encode a focused image of the data code into a phase modulation profile. The complex phase plate structure is fabricated from polylactic acid using fused deposition modeling, a common three-dimensional-printing technique. The design achieves a significantly simplified optical setup, consisting of a 0.14 THz diverging source, the holographic phase plate and a scanning detector, without the need for additional optical elements. The information stored in the data code is an example of a cryptographic private key. Specifically, the private key for a Bitcoin wallet address. Successful retrieval of the encoded information demonstrates a potential use case for terahertz holographic memory, using a storage medium that can be fabricated with consumer-level three-dimensional-printing techniques.

## Introduction

The process of encoding digital information into a holographic image is typically referred to as holographic data storage (HDS). This method of optical memory is achieved by encoding bits into a diffraction, or “interference” pattern embedded within a storage medium. When light of a designated wavelength and phase profile illuminates the storage medium, it is scattered by the diffraction pattern. Through the processes of constructive and deconstructive interference, this creates a holographic image of the stored data, typically in the form of a two-dimensional (2D) bit matrix – a so called “data page”^1–3^. An entire data page can be retrieved instantaneously using a detector array, and many data pages can be encoded into the same storage volume using optical multiplexing techniques, such as phase, frequency or angular modulation of the illuminating light^4–6^.

The upper theoretical limit for volumetric HDS capacity follows a $V/\lambda ^3$ dependence, where *V* is a volume and $\lambda$ is the wavelength of light^7^. The main advantage of HDS is therefore its potential to store large quantities of data in a small physical space, accompanied by fast transfer rates and search capabilities^8^. This is in contrast to the conventional optical storage of compact and digital versatile discs (CDs and DVDs), where data is stored point-by-point as a linear series of bits spread over a surface, and retrieved by optically scanning through each bit sequentially^9,10^.

Despite its clear potential and extensive research history^7,11^, the widespread adoption of HDS has been hindered by several technological challenges. These include simplifying the complex optical setups required to read and write the holographic memory^12,13^, as well as developing suitable materials that can act as reliable and affordable storage media^14,15^. Furthermore, competition from continually advancing solid-state flash memory and cloud storage options challenges the immediate necessity of HDS technology in general.

Nevertheless, the unique qualities of HDS as a non-bit-wise oriented storage method may offer benefits beyond the promise of large data capacities and fast transfer rates. For example, because the data is encoded as a diffraction pattern, which is essentially an optically inverted phase profile of the data-page image, the individual bits that form the data page are not represented by any singular structure within the storage medium. This implies a natural and inbuilt data redundancy. If the diffraction pattern is formed within a mechanically and optically resilient material, it can allow for stable long-term storage that requires no energy to maintain and is naturally robust to local defects and random bit-flip errors. Additionally, because data recovery is an all-optical process, accessing the data does not destroy nor degrade it. Moreover, optical encryption techniques like random-phase encoding can be implemented, which can restrict unauthorised observation of the stored data altogether^16–18^. This makes HDS well suited for archival and data-security applications^19^.

The majority of research into HDS has so far focused on visible radiation with wavelengths of approximately 400-800 nm^1,2^. Longer wavelengths have mostly been neglected because of the inverse and non-linear relationship between data-storage density and optical wavelength. On the contrary, it can be useful to explore longer “non-conventional” wavelengths as a way to test alternative optical arrangements, materials and fabrication methods. The lessons learned may subsequently be reapplied to optimise systems utilising shorter, ‘more practical’ wavelengths, or be directly useful in specialised applications involving short data strings, such as hash outputs or cryptographic keys.

For example, terahertz (THz) radiation, with wavelengths in the range of 0.03-3 mm (10 - 0.1 THz) has optical properties well suited to the refractive qualities and fabrication resolution offered by three-dimensional (3D) printable thermoplastics^20^. Indeed, the use of 3D-printed optical elements such as lenses and diffraction gratings to manipulate and control THz radiation is now a well established technique^21^. In particular, the ability to fabricate complex structures by 3D printing has proven effective in the development of diffractive phase-plate optics that can form holographic images in the THz range^22,23^. This makes the THz frequency range a compelling platform to investigate the practical nuances of computer-generated holographic digital encoding.

In this article, we test the limits of THz beam shaping by demonstrating HDS at terahertz wavelengths using a computer-generated 3D-printed holographic phase plate. The phase plate stores a single data page consisting of a 24 by 24 bit data code and was designed using a modified Gerchberg-Saxton (GS) iterative algorithm^22,24^. By leveraging the freedom of computer-generated design and 3D printing, we achieve a significantly simplified optical setup involving no supporting optics other than the phase plate itself. The data encoded within the holographic bit image is an example of a cryptographic private key generated using Secp256k1 Elliptic Curve Cryptography^25^. Notably, such digital keys play a critical role within the Bitcoin network’s security framework, facilitating digital signatures that enable the verification and authorisation of transactions between so-called “wallet addresses”^26^. The choice of this example highlights a potential practical application from a data security perspective, in which the stored data may be small in numerical size, but can potentially be assigned an arbitrarily high value.

## Results

### Data encoding

To store data holographically, an image of the data must first be converted into a diffraction or phase-modulation profile that can then be encoded into an optical material. To achieve this, we use an optical element called a phase plate, encoded with a computer-generated hologram (CGH)^27^. Fabrication of the complex phase-plate structure is possible using the fused deposition modeling (FDM) 3D-printing technique (see "Methods" section for details).

This approach differs from typical HDS methods used at visible wavelengths, in which the phase-modulation profile is obtained from the interference pattern of recombined coherent light beams. There, the pattern can be directly encoded into an optical material, such as LiNbO$_3$, through photosensitive exposure^2^. The use of a CGH has the benefit of significantly simplifying the optical setup while also allowing flexibility in the storage media material and fabrication process. However, this comes at the expense of intensive computation and slower fabrication, or write, times. Therefore, it is more suitable for so-called “write once, read only memory” applications.

**Figure 1:**
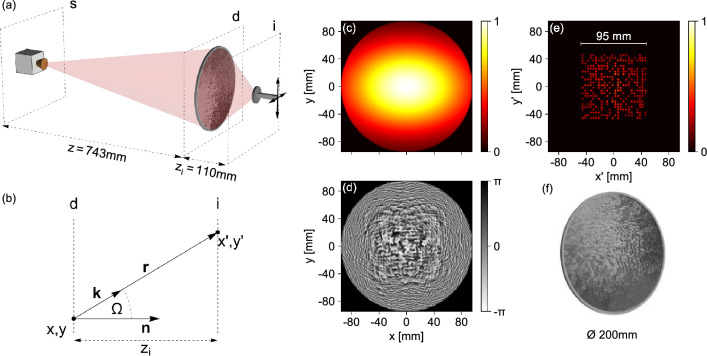
(**a**) Schematic of the experimental setup used to image the holographically stored data matrix. It consists of a source positioned at *s*, a phase plate at the diffraction plane - *d*, and a detector with *xy*-translation measuring the signal at the image plane - *i*. (**b**) Simplified illustration depicting the definitions of vectors $\textbf{r}$, $\textbf{k}$, $\textbf{n}$ and angle $\Omega$ in Eq. (1) between points (*x*, *y*) and $(x',y')$ on the diffraction - *d* and image plane - *i*, respectively. (**c**–**f**) Inputs and outputs of the CGH. The incident beam profile $|E_{(x,y)}^{d}|^2$, presented in arbitrary units (**c**) is modulated by the computer-generated phase-modulation profile (**d**). Here the grey scale represents the relative phase-shift imparted on the incident light. This will produce the target intensity profile at $z_i=110$ mm, $|E_{(x',y')}^{i}|^2$ (**e**). The resulting 3D render of the phase-plate element is shown in (**f**). It consists of the phase-modulation surface with a diameter of 190 mm and a 5 mm wide, 5 mm thick outer ring, giving a total diameter of 200 mm and maximum thickness of 5 mm.

A CGH positioned at a designated diffraction plane that can reproduce a data image at a designated image plane can be obtained using the GS algorithm^24^. The primary inputs for the algorithm are the optical intensity profiles incident at the diffraction and image planes. While the algorithm usually assumes an infinitely-far image plane, we can adapt it for a system with finite geometry (as in Fig. 1a) by incorporating the Huygens principle as a connection between the optical fields at the diffraction and image planes, as demonstrated by Gospodaric et. al.^22^. This principle is summarised in the following form:

1 $$\begin{aligned} E_{(x,y)}^{d}\left( x',y'\right)&=\frac{A_{(x,y)}}{r_{(x,y),(x',y')}}\exp {\left[ -i\textbf{k} \textbf{r}_{(x,y),(x',y')}+i\varphi _{(x,y)}\right] },\nonumber \\ E_{(x',y')}^{i}&=\sum _{(x,y)}E_{(x,y)}^{d}\left( x',y'\right) \left[ 1+\cos \left( \Omega \right) \right] . \end{aligned}$$

Here, $E^{d}$ is defined as a grid of point sources at the diffraction plane. The electric field at the image plane $E^{i}$ is obtained after combining all contributions from $E^{d}(x,y)$ at each coordinate $(x',y')$. As depicted in Fig. 1b, the vector $\textbf{r}$ connects the points in (*x*, *y*) to the points in $(x', y')$, $\textbf{k}$ is the wave vector for each propagating wavelet, and $\Omega$ is the angle between the normal of the diffraction plane and $\textbf{r}$. The simulated intensity profile at the diffraction plane of our setup, derived from imaging the diverging beam of the 0.14 THz radiation source as discussed in the "Methods" section, is shown in Fig. 1c.

As mentioned earlier, the image of the stored data is usually represented as a data page. This involves digitising the data into a 2D bit matrix that can be represented as a plane of bright spots, such that each spot corresponds to a binary unit while the absence of a spot corresponds to a zero^28^. To achieve this, we use the open-source Data Matrix code^29^ to encode our data, which allows us to leverage the built-in error correction as well as its full ASCII capabilities. Using publicly available libraries^30,31^ we encoded a 256-bit (32 byte) private key into a 26 x 26 data matrix code. This is the smallest data matrix size capable of storing our 32 byte private key, including redundancy for error correction. The private key, given in Base64 as a 44 character string, is as follows:

$$\begin{aligned} \mathrm {''6Yc9ecbYfcD7ald4YzOJ9EUyEzA9ph8gvWf8IzqjMmI=''}. \end{aligned}$$

The resulting data page image can be seen in Fig. 1f. The constrained orientation of our experiment allowed us to simplify the Data Matrix by removing the outer-most border of cells (rows and columns numbered 1 and 26) consisting of two solid borders in an L-shape and two borders with alternating dark and light cells. These cells assist in orientation registration and are constant for every Data Matrix. The resulting 2D code was converted into a plane of bright spots and positioned at the center of the target image with an image size of *a*/2, where $a=190$ mm, is the diameter of the phase-modulation surface in the phase-plate optical element. This optical configuration requires the phase plate to act as a converging lens (see "Methods") and takes into account the diffraction limited resolution at the operating frequency. It results in an approximate pixel size of $(a/2)/24\approx 3.96$ mm, which is notably larger than the wavelength of the radiation source $\lambda _0=2.14$ mm, and well within the diffraction resolution limit.

The last parameter required for the modified GS algorithm operating in finite space is the distance between the diffraction plane (phase plate), $z_0$ and the image plane, $z_i$. Using the GS algorithm, we simulated the holographic image for several values of $z_i$. The results showed that for our optical setup, a minimal deviation between the target and simulated holographic image was obtained for an image plane position of $z_i=110$ mm, where we set $z_0=0$.

Incorporating all of the inputs described above, the GS algorithm reaches a convincing convergence of the mean square deviations^22^ after about 500 iterations. Figure 1c–f shows the results of the CGH algorithmic calculation, i.e. a simulation of the intensity profile of the image plane at $z_i$ (Fig. 1e), that results from a phase modulation (Fig. 1d) of the incident beam (Fig. 1c).

To assist in simplifying the practical optical setup and correct for the diverging beam of the source, we combine the calculated phase-modulation profile $\varphi _{\left( x,y\right) }$ (Fig. 1d) and the profile of a Fresnel lens with a focal length equal to 743 mm (see "Methods" for details). Data storage is then achieved by exporting the resulting phase profile as a point cloud $z_{\left( x,y\right) }={\varphi _{\left( x,y\right) } \lambda _0}/{[({\text {Re}}(n)-1)2\pi ]}$ of material thicknesses to be 3D printed, as described in the "Methods" section. A render of the final phase-plate structure is shown in Fig. 1f.

### Data retrieval

The holographically stored data is retrieved by imaging the diffracted radiation pattern at the image plane in the manner depicted in Fig. 1a. By measuring the intensity profile of the holographic image at several distances from the phase plate we pinpointed the ideal experimental image plane approximately 106 mm away from the phase plate. Due to uncertainties in the geometric layout of our setup, we believe it is safe to assume that the image plane position was where it was expected theoretically.

**Figure 2:**
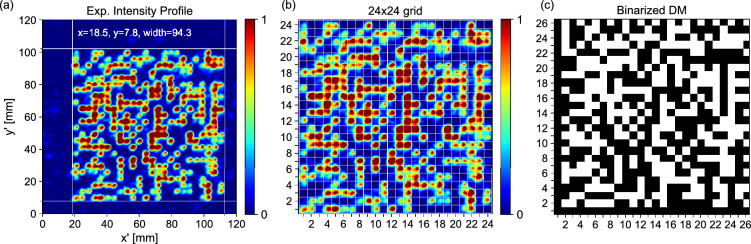
Experimental results and decoding of the holographically stored data page. (**a**) Experimentally measured intensity profile of data page. (**b**) Cropped experimental measurement with overlaid grid for extracting bit values. Reconstructed data matrix (DM) as a result of bit extraction from (**b**) and outer padding with registration border. Note, the intensity peaks in (**a**) and (**b**) correspond to the white bits in (**c**).

As can be seen in Fig. 2a, the result appears as a clear 2D bit matrix, or data page, with an approximate width of 94.3 mm, slightly smaller than expected. The bit edge length in this case is 3.9 mm. We found that the data page can be read rather quickly using a simple sequential search algorithm that runs through different sets of x,y-coordinates, bit widths, intensity thresholds and bit-border widths. The first two parameters of each set define the position of a grid with 24 x 24 cells (as seen in Fig. 2a,b), which are separated by the bit-border width (grey lines in Fig. 2b). The bit-border width truncates the edges of the imaged bits to minimize the effect of bleeding between neighbouring cells. Each cell is then assigned a 0 or 1, depending on whether the mean intensity within the cell is below or above the intensity threshold, respectively.

The resulting 2D grid of binary bits is padded with the registration border pattern as shown in Fig. 2c and imported into standard Data Matrix decoding software. This, somewhat brute-force approach, successfully decoded the imaged data page within seconds of operation. This was also supported by the integrated error correction in the Data Matrix itself, allowing a successful decoding of the data page with up to 20 flipped bits (errors). Using a large bit-border width, resulting in cells that sample only an area of 0.6 mm$\times$0.6 mm at the centre of the 3.9 mm$\times$3.9 mm imaged bits, we were able to extract a data page with 0 errors (examples can be found in the Supplementary Material^32^). Therefore, without the need for redundancy to support error correction in the Data Matrix, in principle it would be possible to encode the 256-bit private key directly as a 16- by 16-bit data page.

## Discussion

Our demonstration of dependable encoding and retrieval of meaningful data from a 3D printed holographic phase plate, in a remarkably streamlined optical configuration, helps to push the boundaries of what can be achieved with 3D printed optics and THz radiation. In doing so, we found that the intersection between the limits of optical resolution, printing detail and data capacity presents an intriguing use case involving the storage of cryptographic keys. In this regard, the demonstration of holographic memory fabricated using consumer-level 3D-printing techniques brings up several interesting points for discussion.

Firstly, it is clear that a major limitation of this approach is the significantly low storage capacity and slow write times. In the demonstrative case of storing a cryptographic key, where long-term stability, security and infrequent access may be desired, these limitations are less of an issue. However, in the general case, it becomes strikingly evident that combining HDS at THz frequencies with a 3D printed storage medium is largely impractical. For example, the theoretical limit of the volumetric storage capacity for holographic data follows a $V/\lambda ^3$ dependence^7^. In the case of a planar hologram, such as our 3D-printed phase plate, the storage capacity follows an $A/\lambda ^2$ dependence, where *A* is the cross-sectional area of the holographic plate^33,34^. Both situations imply a nonlinear improvement in the total storage capacity through the use of shorter wavelengths. While the data storage density of the phase plate presented in this work is $\sim$ 2.03 b/cm$^2$, planar holographic memory created using interferometry and 400 nm light has demonstrated storage densities approaching $\sim$ 2 Gb/cm$^2$^2^. Recent attempts to push the resolution limits of conventional FDM 3D printing has achieved functional printed structures with dimensions in the range of 100 $\mu$m^35^. While a holographic phase plate printed to this resolution could function with radiation frequencies up to 3 THz, data storage density would still remain insignificant when compared to optical wavelengths.

On the other hand, the simplified optics attainable through the flexibility of CGHs and 3D printing may hold potential for enhancing shorter wavelength applications of HDS. In principle, 3D printing techniques can be used to fabricate holographic memory suitable for optical wavelengths, providing that the printing resolution and optical properties of the printed material are sufficient. Fabrication of microscopic diffractive optics has been demonstrated using 2-photon photopolymerization, a form of laser micro fabrication capable of 3D printing with resolutions in the 0.1–1 $\mu$m range^36^. Of course, as the requirement for printing at higher resolutions increases, so does the cost and complexity of the printing technology. Currently, this limits the practicality of fabricating holographic memory in such a way when compared to the interferometry approach. In certain niche use cases, the additional expense incurred to achieve flexibility in demand and scale may indeed be justifiable. One such example being the use of 3D printing within the domain of space exploration^37^.

Another aspect to consider when scaling HDS systems to improve storage density is the relationship between the surface area of the plate for a fixed image size and corresponding data capacity. While the area of the phase plate used for this demonstration was maximised (within the limits of the 3D printer print bed) to collect as much light as possible from the diverging beam, in principle, an equivalent data page image can be achieved with a much smaller phase plate at the expense of a reduced signal to noise ratio.

**Figure 3:**
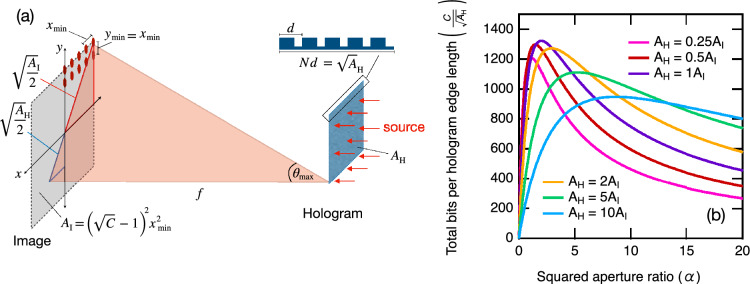
(**a**) Simplified model of a 2D diffraction grating to approximate the resolution limited storage capacity of a holographic phase plate. (**b**) The geometric mean of the data capacity and data density $\left( \frac{C}{\sqrt{A_\text{H}}}\right)$ as a function of the squared aperture ratio ($\alpha$) for different ratios of the hologram and image plane areas. Results are calculated for a wavelength one one hundredth the size of the image plane edge length ($\sqrt{A_\text{I}} = 100\lambda$).

For the sake of discussion, we can approximate the minimal plate size in terms of storage density and optical resolution by using a simplified model in the spirit of Refs.^38,39^ and depicted in Fig. 3. Here we simulate the phase plate with a square diffractive grid composed of $N\times N$ slits spaced *d* apart and producing a grid of spots (or bits) at the image plane. The minimum difference in the phase shift of light emanating from different slits that interfere to produce neighboring peak maxima and minima at the image plane is given by^32,40^,

2 $$\begin{aligned} \Delta \phi = \frac{2\pi }{N}. \end{aligned}$$

Without considering the intensity envelope due to a finite slit width, this results in a diffraction limited resolution for the grating, along the length of the image plane^32^,

3 $$\begin{aligned} \Delta x = \frac{\lambda }{Nd}\frac{x^2+f^2}{f}. \end{aligned}$$

Here, *f* is the distance between the holographic plate with area $A_\text{H}$ and the image plane with area $A_\text{I}$. For points at the centre of the image ($f>>x$), the minimum resolvable displacement reduces to the standard far-field case. However, in the quasi-near-field geometry of our demonstration, the diffraction limited resolution diminishes at the boundaries of the image plane where $f\approx x$. To ensure a uniform bit size with maximum concentration in the image plane, we set the spacing between the bits to a diffraction limited resolution corresponding to the holographic element that contributes to the largest deflection of light (as depicted by $\theta _\text{max}$ in Fig. 3). This gives a minimum bit spacing of,

4 $$\begin{aligned} x_\text{min} = \frac{\lambda }{\sqrt{A_\text{H}}}\frac{\left( \sqrt{\frac{A_\text{I}}{{2}}} + \sqrt{\frac{A_\text{H}}{2}} \right) ^2+\alpha A_\text{I}}{\sqrt{\alpha A_\text{I}}}, \end{aligned}$$

where we define $f^2 = \alpha A_\text{I}$, such that $\alpha$ is analogous to the square of an aperture ratio. The maximum data capacity is then given by,

5 $$\begin{aligned} C = \left( \frac{\sqrt{A_\text{I}}}{x_\text{min}} + 1\right) ^2 = \left[ \frac{1}{\lambda }\frac{A_\text{I}\sqrt{\alpha A_\text{H}} }{\left( \sqrt{\frac{A_\text{I}}{{2}}} + \sqrt{\frac{A_\text{H}}{2}} \right) ^2+\alpha A_\text{I}} + 1\right] ^2. \end{aligned}$$

This expression reveals that for a fixed image area and maximised data capacity, the data storage density ($C/A_\text{H}$) is optimised for a holographic plate area $A_\text{H} = A_\text{I}$ with a focal length $f = \sqrt{2A_\text{I}}$. We can depict this by observing the change in the geometric mean of the data capacity and data density plotted as a function of $\alpha$ for different ratios of the hologram and image plane areas (see Fig. 3). This essentially shows the total data capacity per edge length of the hologram plane, with units of spatial frequency.

Thus, a holographic plate with an area of $\sim$ 95 mm $\times$ 95 mm could in principle reproduce the image of Fig. 2a using 0.14 THz radiation. This would give a slight improvement to the data storage density of the demonstrated phase plate, increasing to $\sim$6.4 b/cm$^2$. A much smaller resolution-limited image, with the same data capacity and optimised for 3 THz radiation, could be produced using a holographic plate with approximate dimensions 3 mm $\times$ 3 mm achieving a data density of $\sim$6.4 kb/cm$^2$. In the context of storing digital keys, a fast access kb/cm$^2$ data density could prove useful given the expected increase in key sizes for next-generation quantum resistant cryptographic protocols^41^. By implementing diffractive multiplexing techniques^42^, these storage capacities could be increased even further. Although, in practise, there are also numerous factors, such as aberrations due to the finite size and imperfect material qualities of the optical elements, which combine to limit the resolution of practical holographic plates^34,43^. These factors must also be considered when determining true data storage capabilities and are beyond the scope of this toy model discussion.

A perhaps unsurprising, yet not entirely obvious consequence of this model, is that without the use of multiplexing techniques, there is no storage density gained by using planar HDS when compared to conventional real-space (“photographic”) encoding of the data matrix^44^. Therefore, one of the primary benefits in using planar HDS technology rather lies in the non-bit-oriented nature of the stored data, where all diffractive components of the holographic plate contribute to all the stored bits. This offers a natural layer of data redundancy and encryption. For example, even if a significant portion of the phase plate is damaged, all of the stored data may still be recoverable. At the same time, a causal inspection of the phase plate’s holographic phase-profile does not directly reveal the content or size of the stored data. Ideally, only a precise optical measurement can be used to extract the data.

Of course, it should be noted that due to the time-reversal symmetry of the Maxwell equations, the holographic phase-profile itself is only a weak form of encryption. With a well resolved image of the phase profile such as in Fig. 1d, a similar algorithm to the one used to generate the holographic phase profile could be used to reverse engineer the stored image, so long as the optical geometry, source frequency and refractive index of the storage medium were known. Together these properties can make for a large search space, but not an insurmountable one. With, sensible estimations of these values a determined attacker, with moderate computational power, could recover the data page image.

To address this issue, an optically opaque but THz transmissible capping layer could be applied to the phase plate during the printing process. Alternatively, optical encryption techniques, such as random phase encoding, could also be implemented^4^. Random phase encoding is a type of phase-code multiplexing, which randomizes the phase of the incident radiation and produces a hologram from the phase-coded waveform^16,17^. One way to achieve this is to essentially split the encoded data between two phase plates, one being a phase key and the other focusing the data page image. Knowledge of ether of the phase profiles alone does not provide enough information to recover the data by computational inversion. The data could only be retrieved by performing the optical experiment with both plates together.

A final note on the security aspects of THz radiation. Currently, THz sources and detectors are not generally accessible outside of research institutions. While this may detract from the general accessibility of this technique as a method to store sensitive digital keys, it also adds a further level of security. As next generation 6G and 7G technologies emerge, this premise may be flipped.

In summary, we demonstrate holographically stored data at sub-THz frequencies with a computer-generated phase plate fabricated using consumer-level 3D-printing techniques. This innovative approach allowed us to achieve a highly simplified optical setup in which the phase-plate memory unit can be illuminated with a diverging beam and imaged without any other supporting optical elements. The data encoded into the phase plate is an example of a cryptographic private key for a Bitcoin wallet address. While the capacity of stored data is small compared to other common data storage methods, the example use case presented suggests that data security applications might be a good fit for this technology. For such applications, overall data capacity is less important, with more emphasis on long-term data integrity. The ability to fabricate the phase plate on-site and on-demand offers further security by allowing one to verify that the memory unit has not been tampered with during production. Using the same approach outlined in this article, it should be possible to further miniaturize the phase plate and increase practical data capacity by operating at higher frequencies and using different multiplexing approaches.

## Methods

A schematic depiction of the experimental setup is shown in Fig. 1a. It consists of an IMPATT diode emitter producing a diverging 0.14 THz ($\lambda _0=2.14$ mm) beam with $\sim$30 mW of power. A pyroelectric detector on a transverse translating stage was used to measure the intensity profile in the *xy*-plane (normal to the optical axis, *z*) with a step-wise scanning resolution of 0.5 mm. The input bias of the IMPATT diode was amplitude modulated at 13.33 Hz to facilitate lock-in detection, significantly increasing the signal to noise ratio at the detector. The holographic phase-plate element was positioned between the source and detector and was designed to directly focus the diverging beam from the diffraction plane onto an image plane at the position of the detector.

For input into the GS algorithm, the incident intensity profile at the diffraction plane, was determined by measuring the transverse intensity profile of the diverging beam at various distances from its approximated point source at the plane *s* in Fig. 1a. The results were analysed using a 2D asymmetric Gaussian model. This quantified how the beam width along *x* and *y* ($w_x$ and $w_y$) increases with *z*. Within the constraints of our optical setup, we were able to apply a linear model for the three-dimensional spatial dependence of beam amplitude and phase profile with good accuracy. Taking the geometry of the setup and the maximum print-bed size of the 3D printer used for fabrication into consideration, we chose to position the phase plate at $z=743.4$ mm. In this region, the beam exhibited an approximately Gaussian profile, with widths $w_x=137.4$ mm by $w_y=93.5$ mm (see Fig. 1c) and a spherical phase front centered at the intersection of the *z* axis and plane *s*, 743 mm from the diffraction plane.

Details of the data storage process, involving computation of the holographic phase-modulation profile, are covered in the "Results" section. Due to the divergent nature of the incident beam in our setup, the final phase-plate profile is formed from a combination of the holographic phase modulation profile and a diffractive (Fresnel) lens profile. Our GS algorithm requires the incident wave to be collimated and planar. Therefore, informed by the spatial mapping of the diverging beam profile described above, the lens component of the phase modulation was specifically designed with a focal length of 743 mm. This corrects for the non-planar phase front of the actual incident beam, collimating the beam at the point of holographic modulation. The resulting point cloud $z_{\left( x,y\right) }={\varphi _{\left( x,y\right) }\lambda _0}/{[({\text {Re}}(n)-1)2\pi ]}$ of material thickness was exported with 0.33 mm resolution.

To 3D print the phase-plate structure we converted the point cloud into a three-dimensional mesh with a 0.4-mm-thick base layer and a 5-mm-thick outer ring to assist in mechanical stability and physical mounting to the optical bench. The resulting phase-plate mesh was imported as an STL file into a 3D printer slicing program. The PrucerSlicer program was used to prepare the 3D printing instructions with a 0.2 mm layer height and 100% concentric infill. The phase plate was printed from Filamentum “natural” (unpigmented) PLA. At the frequency of our radiation source (0.14 THz), the optical properties of PLA are suitable for phase modulation with a complex refractive index of $\tilde{n} \approx 1.59 + 0.005i$, corresponding to moderate dispersive and reflective qualities, with $\sim 6\%$ absorption and $\sim 5\%$ reflection^32^. The 3D printer was a Pruser MkIII, with a maximum print bed size of 200 x 250 mm and a 0.4 mm brass nozzle. The total print time of the phase plate was 11 hours.

### Supplementary Information

Supplementary Information.

## Data Availability

The datasets used and/or analysed during the current study are available from the corresponding author on reasonable request.
