# Supplementary material for: Encoding terahertz holographic bits with a computer-generated 3D-printed phase plate
Source: Sci Rep. 2024 Mar 6;14:5549. doi: 10.1038/s41598-024-56113-2 (PMC11319338; doi:10.1038/s41598-024-56113-2)
Supplement: Supplementary file 1 — Supplementary Information. [file 41598_2024_56113_MOESM1_ESM.pdf]

Supplementary material: Encoding terahertz holographic bits with a computer-generated 3D-printed phase plate

E. Constable[1], **J. Gospodaric**[1], **A. Pimenov**[1]
[1]TU Wien, Solid state spectroscopy, Vienna, Austria

1 Polylactic acid refractive index

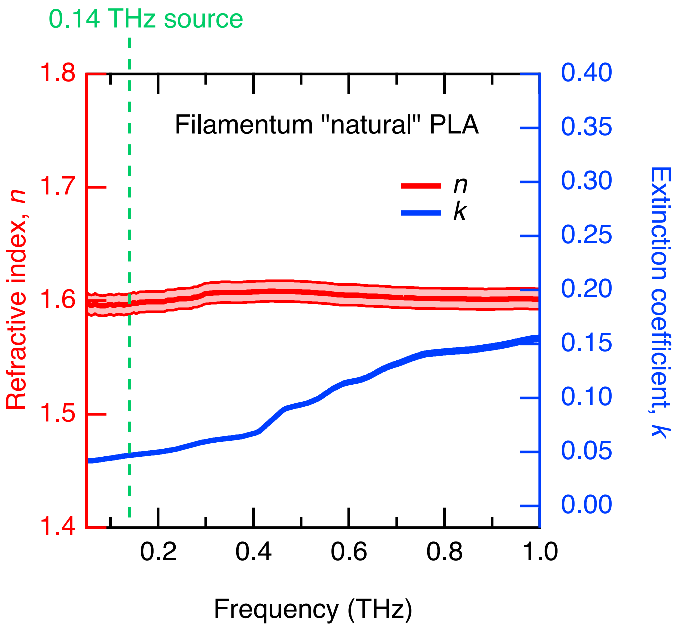

Fig 1 Complex refractive index ($\tilde{n} = n + ik$) for the polylactic acid (PLA) filament used in the construction of the holographic phase plate. Shaded areas give the uncertainty due to sample thickness with a measured value of $d = 2.00 \pm 0.03$ mm. Anomalies in both n and k between 0.3 and 0.8 THz are due to residual water absorption in the optical path. The measurements were performed in transmission through atmosphere on a time-domain terahertz spectrometer utilising photoconductive antennae for the source and detector elements. The sample was 3D printed as a disc with a nominal thickness of 2 mm and a diameter of 10 mm. The print layer height was 0.2 mm with 100% rectilinear infill. The rectilinear infill alternates between 2 orthogonal directions for successive layer depositions, meaning there should be no polarisation dependence in the refractive index of the sample as a result of the layering direction.

2 Bit image truncation in the data retrieval algorithm

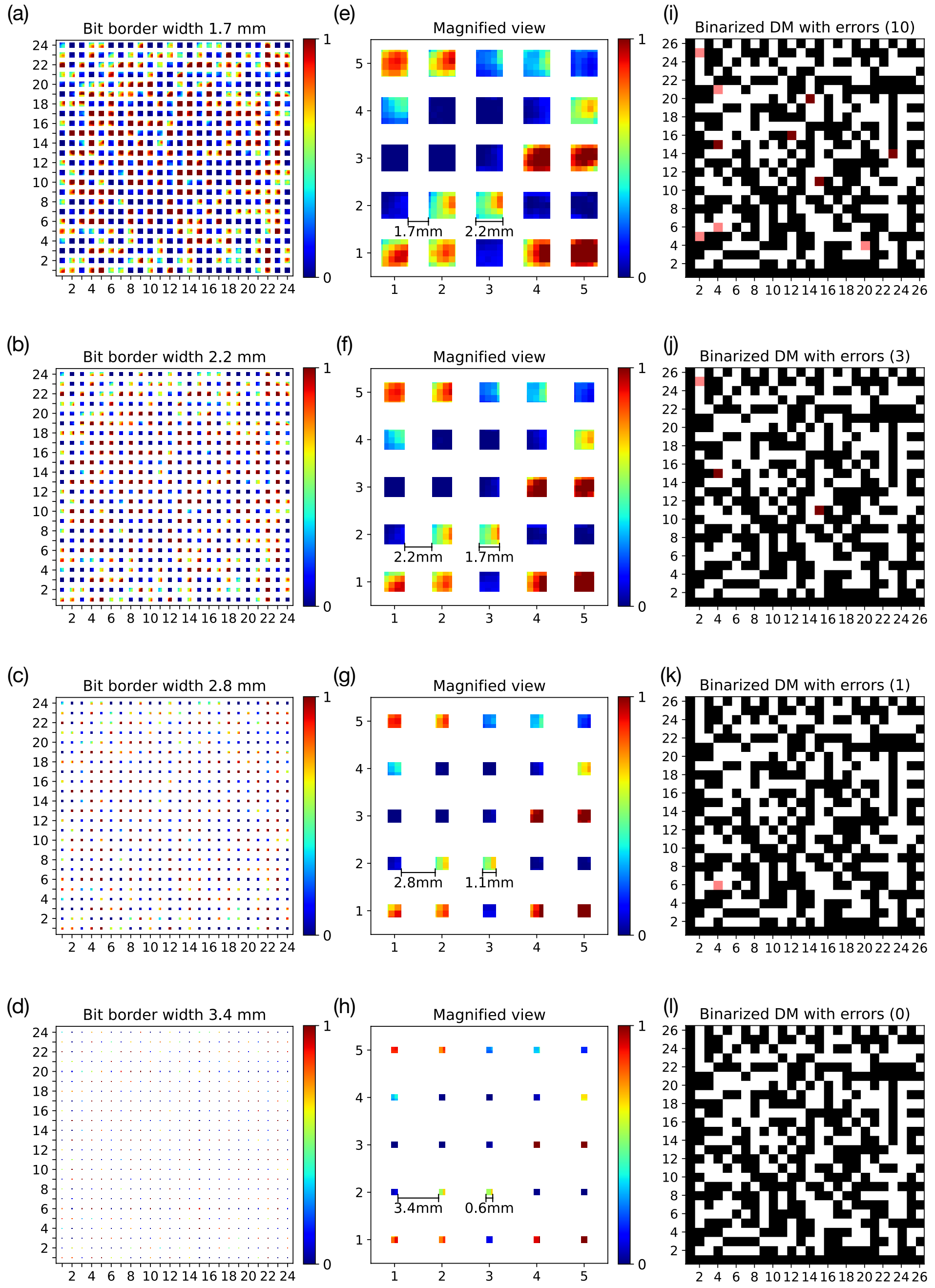

Fig 2 Comparison of the reconstructed data matrix for different bit boarder widths implemented by the data retrieval algorithm. (a-d) Cropped image of the holographic data matrix with overlaid grid for different bit boarder widths of 1.7 mm, 2.2 mm, 2.8 mm and 3.4 mm, respectively. (e-h) Magnified views of the truncated bit images for each bit boarder width. Increasing the boarder width reduces the effects of bleeding between neighbouring bits. (i-l) Reconstructed Data Matrix for the corresponding bit boarder width implementation. Each cell value is obtained by averaging the intensity of each truncated bit image and binarising at a threshold of 0.15 on the normalised scale. Values >0.15 are denoted as white pixels (0s) and <0.15 denoted as black pixels (1s). Erroneous bits are highlighted in red. The largest bit boarder width achieved no errors.

3 Resolution of a simplified diffraction grating

The intensity profile of light diffracted by a grating as depicted in Fig. 3 is given by,[1]

$$I(\phi) = \left(\frac{\sin(\frac{N\phi}{2})}{\sin(\frac{\phi}{2})} \right)^2 I_0, \tag{1}$$

where I_0 is the intensity of the incident light. From this we can deduce that the difference in the phase shift (ϕ) that produces a maximum and one that produces a neighboring minimum is given by,

$$\Delta\phi = \frac{2\pi}{N}. \tag{2}$$

From the geometry of Fig. 3 we can see that the infinitesimal relationship between a change in the phase shift and a change in the angle θ is given by,

$$\frac{d\phi}{d\theta} = \frac{2\pi}{\lambda} d\cos(\theta), \tag{3}$$

or,

$$\begin{aligned} d\phi &= \frac{2\pi d}{\lambda} \frac{f}{\sqrt{x^2 + f^2}} d\theta, \\ &= \frac{2\pi d}{\lambda} \frac{f}{\sqrt{x^2 + f^2}} \frac{dx}{\sqrt{x^2 + f^2}}. \end{aligned} \tag{4}$$

Moving to the discrete approximation and equating Eq. 2 with Eq. 4, we get,

$$\frac{2\pi}{N} = \frac{2\pi d}{\lambda} \frac{f}{x^2 + f^2} \Delta x. \tag{5}$$

This implies a maximum resolving power, for displacement along x, of,

$$\Delta x = \frac{\lambda}{Nd} \frac{x^2 + f^2}{f}. \tag{6}$$

It should be noted that we have neglected the effects of a finite slit width (s), assuming $d >> s$. Thus, we have also neglected the envelope intensity profile resulting from a single slit. The interested reader is directed to standard textbooks on optics, e.g. Reference.[1]

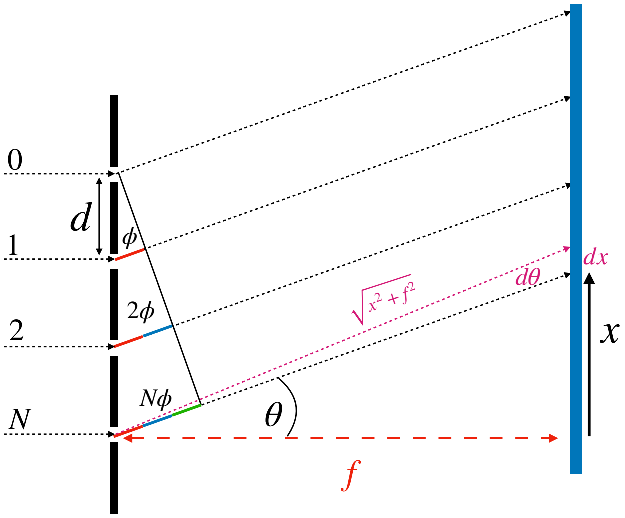

Fig 3 Diagram of diffraction grating with N slits spaced d apart. The incident light is normal to the grating. The phase shift for beams emanating from neighboring slits and arriving at the blue plane on the right hand side is given by $\phi = \frac{2\pi}{\lambda} d \sin(\theta)$, where λ is the wavelength of the light.

References

1 M. Born and E. Wolf, *Principles of optics: electromagnetic theory of propagation, interference and diffraction of light*, Elsevier (2013).
